# SLC37A4‐CDG: Second patient

**DOI:** 10.1002/jmd2.12195

**Published:** 2021-01-06

**Authors:** Matthew P. Wilson, Dulce Quelhas, Elisa Leão‐Teles, Luisa Sturiale, Daisy Rymen, Liesbeth Keldermans, Valérie Race, Erika Souche, Esmeralda Rodrigues, Teresa Campos, Emile Van Schaftingen, François Foulquier, Domenico Garozzo, Gert Matthijs, Jaak Jaeken

**Affiliations:** ^1^ Laboratory for Molecular Diagnosis Center for Human Genetics, KU Leuven Leuven Belgium; ^2^ Centro de Genetica Medica Jacinto de Magalhaes, Centro Hospitalar Universitário de São João Porto Portugal; ^3^ Centro de Referência de Doenças Hereditárias do Metabolismo, Centro Hospitalar Universitário de São João Porto Portugal; ^4^ CNR, Institute for Polymers, Composites and Biomaterials (IPCB) Catania Italy; ^5^ Department of Pediatrics Center for Metabolic Diseases, University Hospitals Leuven Leuven Belgium; ^6^ De Duve Institute, UCLouvain Brussels Belgium; ^7^ Univ. Lille, CNRS, UMR 8576, UGSF, Unité de Glycobiologie Structurale et Fonctionnelle Lille France

**Keywords:** CDG, G6PT1, glycogen storage disease, glycosylation, hepatopathy, SLC37A4

## Abstract

Recently, a disorder caused by the heterozygous de novo c.1267C>T (p.R423*) substitution in *SLC37A4* has been described. This causes mislocalization of the glucose‐6‐phosphate transporter to the Golgi leading to a congenital disorder of glycosylation type II (SLC37A4‐CDG). Only one patient has been reported showing liver disease that improved with age and mild dysmorphism. Here we report the second patient with a type II CDG caused by the same heterozygous de novo c.1267C>T (p.R423*) mutation thereby confirming the pathogenicity of this variant and expanding the clinical picture with type 1 diabetes, severe scoliosis, and membranoproliferative glomerulonephritis. Additional clinical and biochemical data provide further insight into the mechanism and prognosis of SLC37A4‐CDG.

**SYNOPSIS**

Confirmation of a new congenital disorder of glycosylation caused by a heterozygous *SLC37A4* variant that truncates the C‐terminus of G6PT1.

## 1 INTRODUCTION

The *SLC37A4* gene encodes the glucose‐6‐phosphate transporter (G6PT1) responsible for the transport of glucose‐6‐phosphate across the endoplasmic reticulum (ER) membrane.^1^, ^2^, ^3^ In the ER, G6P is cleaved by the ER glucose‐6‐phosphatase to glucose and phosphate and both are exported into the cytosol. This process is critical for the maintenance of normal blood sugar levels and energy metabolism.

In SLC37A4‐CDG, recently reported in one patient, a heterozygous de novo variant (c.1267C>T; p.R423*) on one allele removes the final seven amino acids of the translated protein. This contains the ER retrieval signal and its removal exposes a Golgi retention signal. Mutant G6PT1 is relocated to the Golgi where it disturbs protein N‐glycosylation, causing a congenital disorder of glycosylation (CDG) associated with liver disease and mild dysmorphism.^4^

Intriguingly, biallelic pathogenic variants in *SLC37A4* lead to glycogen storage disease (GSD) 1b [MIM: 232220]. However, in SLC37A4‐CDG, the intact allele is able to fulfil the role required for normal glucose metabolism and therefore no features related to a deficiency of G6P transport are present. This report details another patient with the same heterozygous de novo c.1267C>T; p.R423* variant in *SLC37A4*. Besides liver disease, this patient also presented with type 1 diabetes, severe scoliosis, and membranoproliferative glomerulonephritis.

## 2 MATERIALS AND METHODS

### 2.1 Genetic analysis

Sequence validation of next generation sequencing (NGS) data and segregation analysis were performed by Sanger sequencing of polymerase chain reaction (PCR) products amplified from genomic DNA surrounding exon 11 of the *SLC37A4* gene (ENST00000545985). Amplicons were purified using the PureIT ExoZAP procedure before sequencing with the Big Dye Terminator Cycle Sequencing System v.3.1 (Applied Biosystems) on an ABI PRISM 3100 Genetic Analyzer (Applied Biosystems).

Designation of the heterozygous c.1267C>T; p.R423* variant is based on the coding and polypeptide sequences of the most widely expressed isoform of G6PT1 (Uniprot: *O43826‐1*; RefSeq: NM_001164277.2). This is distinct from isoform 2 (Uniprot: *O43826‐2*; RefSeq: NM_001164278.2) which contains an additional 22 amino acids at position 328 in the polypeptide. According to the ACMG criteria for the classification of pathogenic variants, the variant shows strong evidence of pathogenicity due to the de novo inheritance pattern, with paternity and maternity confirmed.

The patient was investigated, using NGS, as part of a cohort of undiagnosed patients with biomarkers indicative of congenital disorders of glycosylation. This patient was included within this cohort due to a type 2 serum transferrin isoelectrofocusing pattern. Next generation sequencing was performed as follows. Libraries were prepared using the Roche KAPA HyperPrep PCR‐free library preparation kit. Paired‐end 150 bp reads were generated on the Illumina NovaSeq platform. Fastq files were converted to unaligned BAM files prior to upload on Google Cloud platform. Unaligned BAM files were processed according to GATK best practices as in the GATK gatk4‐genome‐processing‐pipeline WLD workflow. Reads were mapped to the reference genome hg38 using BWA mem (version 0.7.15). Picard MarkDuplicates (1.1150) was used for reads deduplication. Base quality score recalibration was performed using GATK BaseRecalibrator and ApplyBQSR (4.0.1.1) before single nucleotide variants and small indels calling with GATK HaplotypeCaller (3.5). Variants annotation used Annovar (October 24, 2019).

### 2.2 Biochemical analyses

Capillary zone electrophoresis (CZE) and transferrin isoelectric focusing (IEF) were performed as described.^5^

### 2.3 Glycan analysis by mass spectrometry

Transferrin was purified from patient's serum by immunoaffinity chromatography on anti‐human transferrin spin columns (Genway Biotech) as previously described.^6^ N‐glycans were released by peptide N‐glycosidase F (PNGase F) digestion and purified by solid‐phase extraction on Hypercarb cartridges. The oligosaccharide mixture was converted in the ammonium form by Dowex 50WX8‐200 cation‐exchange resin, then dried in a centrifugal concentrator and resuspended in a few microliters of 0.1% trifluoroacetic acid before matrix‐assisted laser desorption ionization (MALDI) analysis in negative polarity, using 2′,4′,6′‐trihydroxy‐acetophenone as matrix solution. Mass spectra were acquired in linear negative mode on a voyager STR mass spectrometer (Applied Biosystems, Framingham, Massachusetts).

HEK 293 Cells were treated with 20 μM swainsonine for 24 hours before being sonicated in extraction buffer (25 mM Tris, 150 mM NaCl, 5 mM EDTA and 1% 3‐((3‐cholamidopropyl)dimethylammonio)‐1‐propanesulfonate, pH 7.4) and then dialyzed in 6 to 8 kDa cutoff dialysis tubing in an ammonium bicarbonate solution (50 mM, pH 8.3) for 48 hours at 4°C and lyophilized. The proteins/glycoproteins were reduced and carboxyamidomethylated followed by sequential tryptic and PNGase F digestion and Sep‐Pak purification. Permethylation of the freeze‐dried glycans and matrix‐assisted laser desorption ionization‐time of flight‐mass spectrometry (MALDI‐TOF‐MS) of permethylated glycans were performed as described elsewhere.^7^

### 2.4 Ethics statement

All procedures followed were in accordance with the ethical standards of the responsible committee on human experimentation (institutional and national) and with the Helsinki Declaration of 1975, as revised in 2000 (5). Informed consent was obtained from the patient for being included in the study. Additional informed consent was obtained from the patient for which identifying information is included in this article.

## 3 RESULTS

### 3.1 Patient report

This female was born in 1997 after a normal pregnancy and a caesarean section (pelvic presentation) as the only child of unrelated parents. Weight at birth was 3580 g (P50‐75), length 50 cm (P50) and head circumference 34.5 cm (P25‐50). Apgar score was 8/10. She was admitted at day 36 because of prolonged jaundice (from day 1) and vomiting. Except for jaundice, hepatomegaly (2 cm), and mild splenomegaly, clinical examination was normal. Biochemical screening showed mild microcytic anaemia (Hb 10.7 g/dL), increased serum bilirubin (total 7 mg/dL, direct 4 mg/dL), transaminases (aspartate aminotransferase 522 U/L and alanine aminotransferase (ALT) 494 U/L; normal < 30), activated partial thromboplastin time (aPTT) 38.5 seconds (normal 24.5‐36.5), PT 15.3 seconds (normal 10.6‐12.8), decreased fibrinogen (102 mg/dL; normal 190‐400) and normal albumin, leukocyte, neutrophil and platelet counts. Laboratory investigations were negative for infections like hepatitis A, B and C, HIV, TORCH, and syphilis. Metabolic screening showed normal urinary organic acids, orotic acid and sugars (including reducing sugars), blood ammonia, plasma amino acids, lactate and pyruvate, free and total carnitine, free T4 and thyroid stimulating hormone, α‐fetoprotein, and a normal sweat test. Abdominal ultrasonography showed hepatomegaly, normal bile ducts, and discrete splenomegaly. Ophthalmological, hearing, and cardiac assessments were normal.

Liver biopsy at 4 months showed fibrosis and bile duct proliferation on histology. Jaundice disappeared by this age but hepatic dysfunction (increased transaminases and deficient coagulation) persisted under vitamin K. Psychomotor development was normal as well as a brain magnetic resonance imaging at 2.5 years. Hypoglycemia was never recorded. Although PT normalized, coagulation parameters became progressively worse, with a persistent increase of aPTT (between 50 and 70 seconds).

**FIGURE 1:**
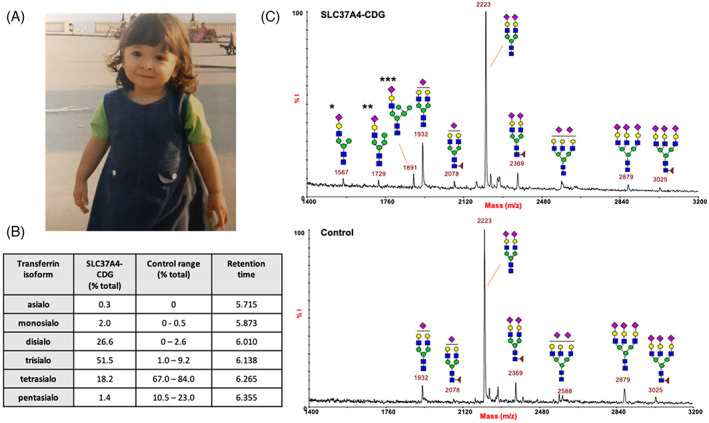
Abnormal glycosylation of serum transferrin in a patient with SLC37A4‐CDG caused by the heterozygous c.1267C>T; p.R423* mutation. A, Photograph of the patient at 3 years of age. B, Capillary zone electrophoresis results, indicating a type 2 pattern. C, matrix‐assisted laser desorption ionization‐time of flight (MALDI‐TOF) mass spectrum (linear mode and negative polarity) of acidic N‐glycans from serum transferrin. Abnormal N‐glycans include: *SiaGalGlcNAcMan3GlcNAc2; **SiaGalGlcNAcMan4GlcNAc2; ***SiaGalGlcNAcMan5GlcNAc2. Symbols represent sugar residues as follows: blue square, *N*‐acetylglucosamine; green circle, mannose; yellow circle, galactose; purple diamond, sialic acid; red triangle, fucose

Meanwhile some minor facial dysmorphism (hypertelorism, saddle nose, hypoplastic nostrils, pointed chin) became evident (Figure 1A) as well as a *pectus excavatum*. The first carbohydrate‐deficient serum transferrin analysis, performed at 2 years, showed a high value of 48.6 % (normal < 1.7). Initially, phosphomannose isomerase deficiency (MPI‐CDG or CDG‐Ib) was suspected and mannose supplementation was tried without success. Subsequently, serum transferrin isoelectrofocusing revealed a type 2 pattern.

During childhood, height and weight remained on the 50th centile. Hepatomegaly (2 cm) persisted and since the age of 4 years the coagulopathy worsened, with easy bleeding in spite of vitamin K therapy. She also showed repeated ear infections treated with tympanic tubes placement at the age of 9. Audiogram at 11 years was normal as were ophthalmological, cardiological, and electroencephalographical examinations.

At 8 years, difficult to control insulin‐dependent diabetes type 1 was diagnosed (anti‐insulin antibodies: 1.2 U/mL [normal < 0.5]; anti‐glutamic acid decarboxylase antibodies: 91.6 U/mL [normal < 1.5]), aggravated since adolescence by irregular compliance. During early adolescence, she developed progressive scoliosis needing surgical correction at 13 years (idiopathic juvenile scoliosis without other bone dysplasia). This happened uneventfully under fresh frozen plasma. By the age of 15, she complained of macroscopic, episodic hematuria, followed by persistent microscopic hematuria and microalbuminuria. Renal biopsy showed histologic findings suggestive of membranoproliferative glomerulonephritis. Development of female sex characteristics started at the age of 10; menarche was achieved at 16 years. Menses are normal without excessive blood loss. She is of normal height and body mass index. She attended regular school with good performance.

Control biochemical screening showed again mild microcytic anemia, increased AST (91 U/L) with normal ALT, aPTT (53.1 seconds), lactate dehydrogenase (260 U/L; normal 135‐225), and alkaline phosphatase (202 U/L; normal 38‐145). Antithrombin (0.22 U/mL; normal 0.7‐1.35), protein C (0.59 U/mL; normal 0.75‐1.25), protein S (0.4 U/mL; normal 0.5‐1.4), factor XI (0.42 U/L; normal 0.7‐1.2), haptoglobin (34 mg/dL; normal 50‐320), transferrin (151 mg/dL; normal 230‐430), and α_1_‐acid glycoprotein (24 mg/dL; normal 55‐140) were decreased. Normal results were found for serum immunoglobulins, ceruloplasmin, bile salts, complement factors, antinuclear antibodies, and antineutrophil cytoplasmic antibodies.

Subsequently, trio exome analysis of the patient and the parents showed the presence of the heterozygous de novo c.1267C>T (p.R423*) *SLC37A4* variant in the patient. This was confirmed with Sanger sequencing.

### 3.2 Glycosylation profile

Analysis of serum transferrin glycosylation was performed using CZE.^5^ A type 2 pattern was identified (Figure 1B). IEF of serum transferrin confirmed the presence of a type 2 pattern (data not shown).

Further investigation of the hypoglycosylated transferrin using MALDI‐TOF mass spectrometry indicated the presence of abnormal hybrid type N‐glycan structures (*m/z* 1567, *m/z* 1729, *m/z* 1891), not found in control serum (Figure 1C). This is in accordance with the report of Marquardt et al on the only other reported patient.^4^

**FIGURE 2:**
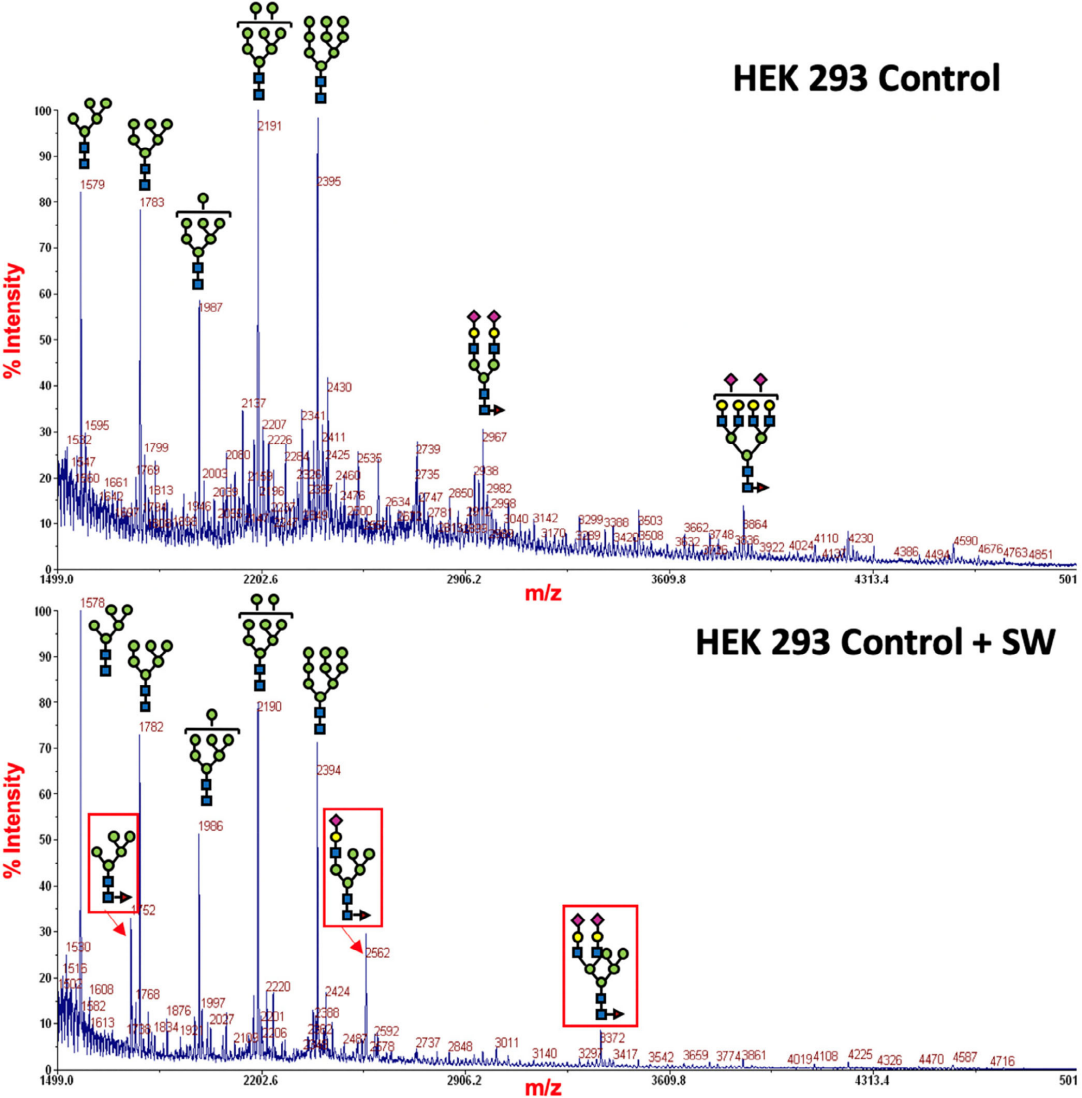
Matrix‐assisted laser desorption ionization‐time of flight‐mass spectrometry (MALDI‐TOF‐MS) spectra of the permethylated *N*‐glycans from HEK control cells following swainsonine treatment. Only the representative glycan structures are shown. Those resulting from swainsonine treatment are highlighted in a red square. Symbols represent sugar residues as follows: blue square, *N*‐acetylglucosamine; green circle, mannose; yellow circle, galactose; purple diamond, sialic acid; red triangle, fucose. Linkages between sugar residues have been removed for simplicity

These findings also correlate with previously published high pressure liquid chromatography and MALDI‐TOF data by Butler et al^8^ on the same patient before a diagnosis had been established. They identified similar abnormal plasma transferrin and IgG glycans. These abnormal glycans consisted of pentamannosyl hybrid type glycans (eg, Man_5_GlcNAc_2_), with varying lengths of the α1‐3‐linked antenna. This points toward a potential deficiency of the Golgi‐localized mannosidase enzymes α‐mannosidase II and α‐mannosidase IIx, responsible for the cleavage of terminal α1‐3 and α1‐6 mannose residues. Indeed, incubation of HEK 293 cells with the Golgi α‐mannosidase II inhibitor swainsonine^9^ also induced the formation of hybrid‐type N‐glycans (Figure 2).

## 4 DISCUSSION

In the present patient, extensive investigations were carried out over a period of almost 20 years. The heterozygous p.R423* variant in *SLC37A4* had already been identified in whole exome sequencing data but initially discarded due to the perceived unlikeliness of haploinsufficiency of the G6PT1 transporter to lead to a CDG. A diagnosis was eventually provided by connecting the presence of this variant with biochemical studies by Marquardt et al on another patient with the same variant and a similar clinical presentation.^4^

Pathogenic biallelic variants in *SLC37A4* affect the function of G6PT1 and prevent the entrance of glucose‐6‐phosphate into the ER causing GSD 1b. This usually causes early onset hypoglycemia and hepatomegaly as well as other metabolic abnormalities linked to hypoglycemia (such as lactic acidosis) and neutropenia.^3^ However, in SLC37A4‐CDG it appears the wild‐type allele is still able to sufficiently perform its normal function in the ER. Instead, glycosylation abnormalities lead to a CDG characterized mostly by hepatic dysfunction, with some dysmorphism and skeletal abnormalities. In the present patient, type 1 diabetes and membranoproliferative glomerulonephritis were also present. Some similarities between GSD 1b and SLC37A4‐CDG can be noted, such as distinct facial features and hepatic dysfunction; however, these are unlikely to be due to a shared pathomechanism, and the classical metabolic symptoms of GSD 1b are not present.

Marquardt et al revealed that the heterozygous p.R423* variant leads to a functional form of the G6PT1, but one that is mislocalized from the ER to the Golgi membrane. This conclusion was supported by the earlier findings of Chen et al who showed that stop mutations later than p.K420* led to normal expression of the active protein, but if the protein was truncated further upstream, expression was reduced.^10^

Marquardt et al^4^ reported increased abnormal high mannose and hybrid‐type transferrin glycans, quite specific findings, and unusual within the field of CDG. We were able to confirm the presence of these abnormal N‐glycan structures in this second SLC37A4‐CDG patient using MALDI‐TOF, in particular, abnormal monosialylated Man_3_, Man_4_, and Man_5_ N‐glycans. This was also found in previous data from the present patient, published by Butler et al.^8^

It is possible that these abnormal glycan structures result from a deficiency of Golgi α‐mannosidase II activity. Butler et al^8^ performed mutation analysis of *MAN2A1* and *MAN2A2* but did not identify any pathogenic variants. These hybrid‐type glycans were also present in HEK 293 cells incubated with swainsonine, a Golgi α‐mannosidase II inhibitor. Accordingly, similar glycan profiles have also been identified in a mouse model of MAN2A1/MAN2A2 deficiency^11^ and in a MAN2A1‐deficient HEK 293T cell line.^12^

In addition, the presence of significant levels of Man_5_ asialo‐N‐glycans in prior work by Butler et al,^8^ as well as SiaGalGlcNAcMan_3_GlcNAc_2_ identified here, could indicate deficiency of β‐N‐acetylglucosaminyltransferase I (GnTI) or other glycosyltransferases. Indeed, it is likely that the action of several Golgi N‐glycan processing enzymes is disrupted. It is difficult, however, to assign a specific mechanism for a reduction in Golgi mannosidase II or glycosyltransferase activity. Marquardt et al suggested that this could be due to the import of G6P into the Golgi apparatus, facilitated by the mislocalized, but active, G6PT1 protein. It was suggested that without the phosphatases required for hydrolysis, G6P may accumulate, thereby disrupting the microenvironment required for proper glycan processing. However, the transport action of G6PT1 is not energy‐dependent. This means that G6PT1 is able to bring the balance of G6P to an equilibrium across a membrane, but unable to concentrate it above the levels found in the cytoplasm.^13^ It is also unclear how raised concentrations of G6P in the Golgi would directly lead to diminished activity of mannosidases, or other enzymes.

Other hypotheses should also be considered. For example, perhaps other aspects of Golgi homeostasis are disrupted, affecting cofactors required for mannosidase II activity, or the activity of other glycosyltransferases. Indeed, there are multiple CDG caused by the disruption of Golgi cofactor homeostasis.^14^, ^15^ In addition, it is possible that the presence of G6PT1 at the Golgi membrane interferes directly with the conformation, oligomerization, or localization of N‐glycan processing enzymes.

The patient reported by Marquardt et al showed a predominantly hepatic disease, with some dysmorphism but without psychomotor or growth abnormalities. A striking similarity in facial features should be noted. The present patient showed in addition type 1 diabetes, severe scoliosis, and membranoproliferative glomerulonephritis. Interestingly, some of the additional symptoms, for example, type 1 diabetes, but in particular the membranoproliferative glomerulonephritis, might be associated with the unusual glycan profile of the patient. Indeed, the observed increase in terminal mannose residues mimics the glycans commonly expressed by lower eukaryotes and prokaryotes. These altered self‐N‐glycans can trigger an innate immune response through binding of lectin receptors. Over time, chronic activation of the innate immune response can induce autoimmune disease. Indeed, Golgi α‐mannosidase II‐deficient mice develop glomerulonephritis based on sensing of altered self‐glycans by the innate immune system.^16^

Additional patients will reveal whether the type 1 diabetes, severe scoliosis, and membranoproliferative glomerulonephritis in our patient are part of the syndrome or whether these are coincidental findings. Also, it is not excluded that the patient reported by Marquardt et al will develop these symptoms at a later age, since this patient was 12 years old at last examination, 10 years younger than the patient reported here. Finally, this study reiterates the continued importance of biochemical phenotyping in the genomic era.

## CONFLICT OF INTEREST

The authors declare no potential conflict of interest.

## AUTHOR CONTRIBUTIONS

Matthew P. Wilson, Elisa Leão‐Teles, Luisa Sturiale, Daisy Rymen, Erika Souche, and Jaak Jaeken wrote the manuscript. Matthew P. Wilson, Daisy Rymen, Liesbeth Keldermans, Valerie Race, Erika Souche, and Gert Matthijs performed genetic analysis. Luisa Sturiale, François Foulquier, and Domenico Garozzo performed biochemical investigations and research. Dulce Quelhas, Elisa Leão‐Teles, Esmeralda Rodrigues, Teresa Campos, and Jaak Jaeken performed clinical investigation and assessment. Emile Van Schaftingen and François Foulquier provided additional intellectual input. Gert Matthijs and Jaak Jaeken supervised research.
